# An Analysis of Malpractice Litigation and Expert Witnesses in Plastic Surgery

**Published:** 2017-09-28

**Authors:** Paul J. Therattil, Stella Chung, Aditya Sood, Mark S. Granick, Edward S. Lee

**Affiliations:** Division of Plastic and Reconstructive Surgery, Department of Surgery, Rutgers New Jersey Medical School, Newark

**Keywords:** plastic surgery, medical malpractice, malpractice litigation, academic productivity, expert witness

## Abstract

**Objective:** Expert witness testimony is crucial for juror decision making. The goals of this study were to examine the trends in malpractice litigation in plastic surgery and to examine the characteristics of expert witnesses in litigation. **Methods:** The Westlaw legal database was queried for jury verdict and settlement reports related to plastic surgery cases from 2009 to 2015. Cases were examined for expert witness testimony, procedure performed, alleged injury, cause of action, verdict, and indemnity payments. **Results:** Ninety-three relevant cases were examined. Mean plaintiff award was $1,036,469, whereas mean settlement was $633,960. The most commonly litigated procedures involved breast surgery (34.4%), liposuction (18.3%), and body contouring (14.0%). Cases involving body contouring (risk ratio [RR] = 1.48; 95% CI, 1.04-2.10) were more likely to result in favor of the defendant, whereas cases involving breast surgery (RR = 0.27; 95% CI, 0.13-0.57) were more likely to result in favor of the plaintiff (*P* < .05). Cases in which there was claimed pain (RR = 1.22; 95% CI, 1.01-1.48) or emotional distress (RR = 1.38; 95% CI, 1.11-1.70) were more likely to result in favor of the plaintiff (*P* < .05). The party of a lawsuit was more likely to win the case if its expert witness was a plastic surgeon (*P* < .05). **Conclusion:** Plastic surgery litigation tends to favor defendants. Most litigation involves breast surgery, liposuction, and body contouring. The type of procedure and alleged claim affect case success. Parties with a plastic surgeon as an expert witness tend to be more successful in litigation.

The rate of malpractice litigation continues to increase yearly. Plastic surgeons are at especially high risk of facing malpractice claims, with recent studies demonstrating that the proportion of plastic surgeons facing malpractice claims each year is 13%. The only specialties with higher rates of claims were neurosurgery (19%), cardiovascular/thoracic surgery (18%), general surgery (15%), and orthopedic surgery (14%). In comparison, nonsurgical specialties had claim rates ranging from 2% to 9%.^1^

Expert witnesses serve as a critical part of the litigation process in medical malpractice cases, providing insight into the judge and jurors who may be unfamiliar with both the medical and specialty-specific aspects of the case. The concept of the expert witness within a field such as plastic surgery, where outcomes might be considered more subjective than in other surgical fields, has been a point of debate. Some have gone so far as to suggest having anonymous physician surveys to replace plaintiff expert witness testimony because of the high stakes and pressure to perform for paying clients.^2^

While subjective experiences and various advisements about legal liability and patient selection have been published in the past, there is a lack of analytical literature regarding medicolegal cases within plastic surgery.^3^^-^5 The goals of this study were to examine the trends in malpractice litigation in plastic surgery, as well as to examine the characteristics of expert witnesses in plastic surgery litigation.

## METHODS

The Westlaw legal research database (Thomson Reuters, New York, NY) was queried for all publicly available state and federal court reports regarding plastic surgery malpractice litigation. The Westlaw database is a source for legal experts to obtain information regarding state and federal cases. Jury verdicts and settlement reports were obtained by using the search terms “medical AND malpractice AND plastic AND surgery OR surgeon” for a time period from 2009 to 2015. Cases were excluded if they were duplicate cases, unrelated to plastic surgery, did not involve a clinician, had anonymous defendant(s), or involved cross-complaints. Cases were examined for year, geographic location, procedure performed, alleged injury, reason for litigation, verdict, indemnity payment, defendant, and expert witness testimony.

Characteristics about the defendants and expert witnesses were obtained from institutional Web sites. Board certification status of surgeons was confirmed at the Web site of *The American Board of Plastic Surgery* (www.abplsurg.org). The Scopus database (www.scopus.com) was queried by surgeon name to determine their current *h*-index. Those surgeons with common names often had multiple authors populated upon query of the Scopus database. The previous and current affiliations of each surgeon were cross-checked with information from the institution's Web site to verify that publications were being attributed to the correct author. When necessary, the field of the publications in question was also verified.

Analysis of cases was performed using analysis of variance. To compare *h-*indices of expert witnesses, nonparametric statistical analysis was performed using Mann-Whitney *U* tests and Kruskal-Wallis tests as indicated. Multivariate regression analysis was performed to determine the significance controlling for independent variables. All statistical analysis was performed using Stata/MP 13.0 (StataCorp LP, College Station, Tex). Threshold for statistical significance was set at *P* < .05 with utilization of the Bonferroni correction method. As per the institutional review board of Rutgers New Jersey Medical School, this study qualifies as nonhuman subject research, as no health information is disclosed by the Westlaw database.

## RESULTS

Our search of the Westlaw database from 2009 to 2015 yielded 165 results. Of these verdict and settlement reports, 93 met the inclusion criteria and were included for final analysis. In this set of cases, 61 resulted in favor of the defendant (65.5%), 22 resulted in favor of the plaintiff (23.7%), and 10 resulted in settlement (10.8%).

Of those cases that resulted in favor of the plaintiff, the mean award was $1,036,469 (range, $10,697-$4,500,000; median = $462,500). Of those cases that resulted in settlement, the mean award was $633,960 (range, $29,000-$1,876,637; median = $550,000).

By state, California (26.9%), New York (18.3%), and Massachusetts (7.5%) had the highest number of legal cases related to plastic surgery in our series. Other highly populated states represented in our series that had lower numbers of cases were Texas (1.1%), Florida (5.4%), Illinois (4.3%), Pennsylvania (5.4%), Ohio (2.1%), Georgia (1.1%), and Michigan (3.2%).

**Figure 1:**
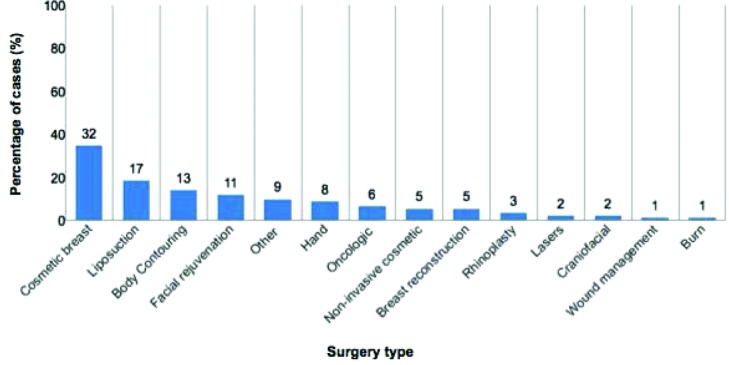
Litigated plastic surgery cases by surgery type (n = 93).

**Figure 2:**
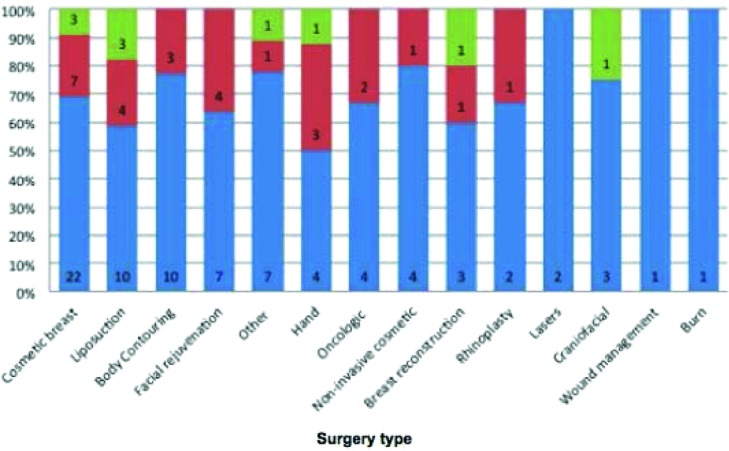
Litigated plastic surgery cases by type and outcome (n = 93) (*green* = settled, *red* = in plaintiff favor, *blue* = in defendant favor).

By procedure type, 32 cases involved cosmetic breast (34.4%), 17 liposuction (18.3%), 13 body contouring (14.0%), 11 facial rejuvenation (11.8%), 8 hand (8.6%), 6 oncological management (6.5%), 5 noninvasive facial cosmetics (5.4%), 5 breast reconstruction (5.4%), 3 rhinoplasty (3.2%), 2 craniofacial (2.2%), 2 laser (2.2%), 1 burn (1.1%), and 1 wound management (1.1%). Nine cases could not be classified (9.7%) (Fig 1). Cases involving body contouring (risk ratio [RR] = 1.48; 95% CI, 1.04-2.10) were more likely to result in favor of the defendant, whereas cases involving breast surgery (RR = 0.27; 95% CI, 0.13-0.57) were more likely to result in favor of the plaintiff (*P* < .05) (Fig 2).

**Figure 3:**
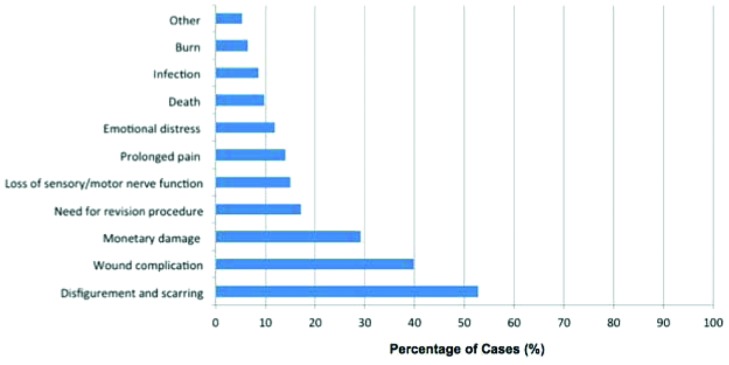
Litigated plastic surgery cases by plaintiff claim (n = 93).

Of the claims made by plaintiffs, 49 included disfigurement (52.7%), 37 for physical injury (39.8%), 27 for monetary damage (29.0%), 16 with need for additional surgery (17.2%), 14 for dysfunction (15.1%), 13 for pain (14.0%), 11 for emotional distress (11.8%), 9 for death (9.7%), 8 for infection (8.6%), and 6 for burns (6.5%). Five other cases could not be classified (5.4%) (Fig 3). Cases in which there was claimed pain (RR = 1.22; 95% CI, 1.01-1.48) or emotional distress (RR = 1.38; 95% CI, 1.11-1.70) were more likely to result in favor of the plaintiff (*P* < .05).

**Figure 4:**
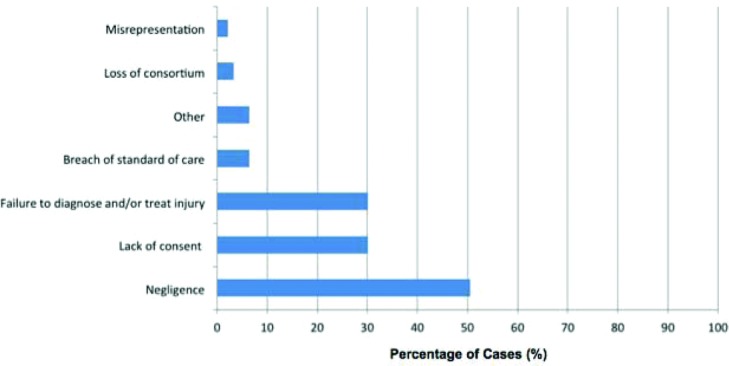
Litigated plastic surgery cases by alleged cause of action (n = 93).

Categorized by cause of action, 47 cases involved negligence (50.5%), 28 lack of informed consent (30.1%), 28 failure to diagnose or treat injury (30.1%), 6 breach of standard-of-case (6.5%), 3 loss of consortium (3.2%), 2 misrepresentation (2.1%), 2 breach of contract (2.1%), 2 surgical error (2.1%), 1 retained foreign body (1.1.%), and 1 wrong procedure (1.1%). One case could not be classified (1.1%) (Fig 4). The degrees of causes of action (primary vs secondary) could not be determined from the data set in these cases.

There were 177 expert witnesses who testified in our 93 cases: 85 on behalf of the plaintiff side and 92 on behalf of the defendant. Of these expert witnesses, 92 were plastic surgeons (52.0%), of which 42 testified on behalf of the plaintiff and 50 testified on behalf of the defendant. Defendant expert witnesses had significantly higher *h*-indices (8.68 ± 1.40) compared with those testifying on behalf of plaintiffs (4.69 ± 1.00) (*P* < .05). Defendant expert witnesses tended to have lower rates of board certification (84.0% vs 95.2%) (*P* = .10) and were more often full-time academic faculty (12.0% vs 9.5%) (*P* = .70), but these differences were not significant. The party of a lawsuit was more likely to win the case if its expert witness was a plastic surgeon (*P* < .05).

Subgroup analysis of cases in which plastic surgeons served as expert witnesses on both sides of the case revealed similar results, with those testifying on behalf of defendants had significantly higher *h*-indices (7.83 ± 1.50) compared with those testifying on behalf of plaintiffs (4.12 ± 1.10) (*P* < .05). Defendant expert witnesses tended to have higher rates of board certification (82.9% vs 64.7%) (*P* = .08) and were more often full-time academic faculty (14.6% vs 5.9%) (*P* = .20), but these differences were not significant. Of this subgroup of plastic surgeons, 24% of defendant expert witnesses and 11.9% of plaintiff expert witnesses testified in multiple cases in our series and always testified for the same side.

## DISCUSSION

Our case series demonstrates that a majority of the recent cases involving plastic surgery are ruled in favor of defendants (65.5%). More than a tenth of cases are settled. The regional distribution of these cases is particularly interesting. The highest proportion of cases in our series came from states with relatively high tort costs, or litigation risk, and weak tort laws (California, New York, Massachusetts, and Pennsylvania).^6^ Although these are relatively populous states, other highly populous states with better tort reform tended to represent much smaller proportions of cases in our series (Texas, Ohio, Georgia, and Michigan).

Prior studies have demonstrated that often the specialties with the highest rates of yearly malpractice claims are not necessarily those with the highest payments to plaintiffs.^1^ For example, neurosurgeons have the highest yearly rate of malpractice claims (19%) but not the highest mean indemnity payment ($344,811). In comparison, pediatricians only have a 5% yearly rate of malpractice claims but the highest mean indemnity payment ($520,924). The mean and median payments to plaintiffs were $1,036,469 and $462,500, respectively, in our series of plastic surgery cases. Interestingly, this was more than 5 times previously reported values in plastic surgery.^1^ These differences may be due to case selection bias in our study, although there is a chance that previous studies are truly underestimating the size of damages in plastic surgery malpractice. Special care should be taken in encounters in the emergency department specifically, as previous studies have demonstrated that these cases have the highest average indemnity payments compared with those that originate in the operating room.^7^

A majority of cases in our series involved nonreconstructive breast surgery, liposuction, and body contouring. This is commensurate with the prevalence of these procedures performed each year: the top cosmetic procedures in plastic surgery in 2013 were liposuction, breast augmentation, blepharoplasty, and abdominoplasty.^8^ In our series, breast surgery cases were more likely to result in favor of the plaintiff overall. It has previously been shown that within cosmetic breast surgery, cases involving alleged negligent misrepresentation and fraud had nearly 2 times greater chance of outcome in favor of the plaintiff.^9^ Thus, although surgeons entering litigation involving cosmetic breast surgery may be more likely to lose, the cause of action alleged should be considered.

Cases involving body contouring in our series were more likely to result in favor of the defendant. It has been demonstrated previously that within body contouring, cases with younger age of plaintiff (36 vs 44 years of age) and claimed disfigurement or iatrogenic injury were more likely to result in outcome in favor of the plaintiff.^10^ Therefore, entering litigation involving body contouring has a higher likelihood of success for the surgeon, although the age of the plaintiff and claim should be considered.

There were a relatively small number of hand cases in our series—only 8.6% of all cases. In general, there is literature lacking with regard to legal liability in hand and microsurgery. A single-institution retrospective study demonstrated that there is a small risk of claims with regard to digital replant procedures (3%), with all claims being dropped eventually. Most claims with regard to replantation actually were related to decisions *not* to replant a digit.^11^

Although blepharoplasty is now the third most common cosmetic procedure performed, there were relatively few cases in our series. This may be a result of delay in reporting, as the increase in number of blepharoplasties performed has been a recent trend. With oculoplastic procedures including blepharoplasty and blow lift, previous studies have shown that although verdicts tended to favor defendants, those cases alleging blindness, cranial nerve injury, or permanent deficit were more like to result in payment to the plaintiff.^12^

Other than reconstructive breast surgery, only 2 cases in our series involved true reconstructive procedures. Both procedures involved radial forearm flaps for upper extremity reconstruction, with alleged negligence resulting in reduced hand function. Both cases resulted in favor of the defendant. Previous studies have shown that plastic surgeons who are deemed by their institution to be “reconstructive” plastic surgeons are at 5 times more likely to be at high risk for malpractice claims than other plastic surgeons.^13^ This could not be replicated in our case series, as a majority of cases were to be cosmetic.

Previously, “lack of informed consent” has been noted to be the most common alleged cause of action in facial plastic surgery procedures.^14^ In addition to “lack of informed consent,” which was the cause of action in nearly one third of cases in our series, negligence (50.5%) and failure to diagnose or treat injury (30.1%) were the other most common causes of action. While disfigurement, physical injury, and monetary damage were the most common claims, pain and emotional distress were significant predictors of plaintiff victory in our series. In contrast, a previous study of legal liability in body contouring found that disfigurement was a significant predictor of plaintiff victory.

The American Society of Plastic Surgeons (ASPS) Code of Ethics declares that its member surgeons are obligated to testify as expert witnesses when appropriate. However, the situation in the United States is unique in comparison with that in other countries where expert witnesses are attached to the court. In the United States, opposing sides in a trial hire and pay their own expert witnesses.^15^ Therefore, there is an inherent potential bias in a malpractice trial with regard to expert testimony. Many have advocated for reform of this system.^16^ It has been recommended by some that physician expert witnesses serve both the plaintiff and defendant sides in various cases to minimize this potential for bias, but it is often the opposite that occurs, with expert witnesses serving the same side for every case in which they take part.^17^^,^^18^ We found a similar trend in our series, where the expert witnesses serving in multiple cases always served the same side. The ASPS Code of Ethics does not address this nuance, and perhaps plastic surgeons serving as expert witnesses require more guidance on this topic.

It has been demonstrated in previous studies that there may be a disparity between the credentials of plaintiff and defendant expert witnesses in medicolegal cases. Therefore, part of this study sought to determine whether these differences exist in plastic surgery malpractice litigation.^18^ Our study demonstrated that expert witnesses serving the defense are generally more highly credentialed than those serving plaintiffs. The *h*-index is one method to measure academic productivity that has been recently validated in plastic surgery.^19^ In cases in which there were plastic surgeons serving as both plaintiff and defendant expert witnesses, the defendant expert witness was significantly more academically productive with a mean *h*-index of 7.83 (vs 4.12 for plaintiff expert witness). Although a defendant expert witness tended to have a higher rate of board certification (82.9% vs 64.7%) and tended to be a full-time academic faculty (14.6% vs 5.9%), these data were ultimately not statistically significant. Interestingly, only just over half of the expert witnesses for plastic surgery litigation in our series were actually plastic surgeons. While this may be acceptable depending on the alleged claim and cause of action, there would certainly be cases in which only having a plastic surgeon testifying would be appropriate. In fact, sides with plastic surgery expert witnesses were more likely to win in our series.

It is important to note that the Westlaw database is limited in that it is not comprehensive—lawyers, or others in the law profession, submit cases to the database voluntarily. This should be considered when interpreting the results of this study and is an obvious weakness. The lack of viable alternatives, however, likely explains the lack of published studies on the subject.

Our series of malpractice cases in plastic surgery reveals a number of interesting trends. States with weak tort reform may have more cases filed than states with stronger tort reform. In addition, payments to plaintiffs from successful plastic surgery claims may be much larger than were initially thought. The type of procedure performed and claim purported appear to affect the likelihood of victory for the plaintiff. Body-contouring cases appear more likely to result in favor of the defendant, whereas breast cases appear more likely to result in favor of the plaintiff. Claims of pain and emotional distress also appear more likely to result in favor of the plaintiff. With regard to expert testimony, sides with plastic surgeons serving as expert witnesses are more likely to win. Furthermore, when plastic surgeons serve as expert witnesses for both sides, the defense's witness tends to be more highly qualified.
